# The decision to delivery interval in emergency caesarean sections: Impact of anaesthetic technique and work shift

**DOI:** 10.12688/f1000research.13058.2

**Published:** 2017-12-11

**Authors:** Anette Hein, David Thalen, Ylva Eriksson, Jan G. Jakobsson

**Affiliations:** 1 Department of Anaesthesia & Intensive Care, Institution for Clinical Science, Karolinska Institutet, Danderyds University Hospital, Stockholm, Sweden

**Keywords:** Caesarean section, anaesthesia, time factors

## Abstract

**Background:** One important task of the emergency anaesthesia service is to provide rapid, safe and effective anaesthesia for emergency caesarean sections (ECS). A Decision to Delivery Interval (DDI) <30 minutes for ECS is a quality indicator for this service. The aim of this study was to assess the DDI and the impact of chosen anaesthetic technique (general anaesthesia (GA), spinal anaesthesia (SPA) with opioid supplementation, or “top-up” of labour epidural analgesia (tEDA) with local anaesthesia and fentanyl mixture) and work shift for ECS at Danderyds Hospital, Sweden.

**Methods:** A retrospective chart review of ECS at Danderyds Hospital was performed between January and October 2016. Time between decision for CS, start of anaesthesia, time for incision and delivery, type of anaesthetic technique, and time of day, working hours or on call and day of week, Monday – Friday, and weekend was compiled and analysed. Time events are presented as mean ± standard deviation. Non-parametric tests were used.

**Results:** In total, 135 ECS were analysed: 92% of the cases were delivered within 30 minutes and mean DDI for all cases was 17.3±8.1 minutes. GA shortened the DDI by 10 and 13 minutes compared to SPA and tEDA (p<0.0005). DDI for SPA and tEDA did not differ. There was no difference in DDI regarding time of day or weekday. Apgar <7 at 5’ was more commonly seen in ECS having GA (11 out of 64) compared to SPA (2/30) and tEDA (1/41) (p<0.05).

**Conclusion:** GA shortens the DDI for ECS, but the use of SPA as well as tEDA with opioid supplementation maintains a short DDI and should be considered when time allows. Top-up epidural did not prolong the DDI compared to SPA. The day of week or time of ECS had no influence on the anaesthesia service as measured by the DDI.

## Introduction

There are around 110,000 births annually in Sweden, and the national statistics shows a trend for an increasing number of caesarean sections (CS). In 2014, 17% of all births in Sweden were CS ^i^. CS may be divided into emergency and elective procedures. Emergency CS (ECS) are commonly defined as follows: to be performed within an adequate time frame to avoid negative effects on neonate and/or mother, while elective are performed where there is no time constrain. Lucas four graded scale categorize CS by degree of urgency, as follows: 1) immediate threat to life of woman or foetus; 2) maternal or foetal compromise that is not immediately life-threatening; 3) needing early delivery but no maternal or foetal compromise; and 4) at a time to suite patient and maternity team ^1^. Dupuis suggested a coloured system to distinguish grade of emergency, to facilitate the communication, thus facilitating the process and shorten the DDI ^2^.

Need for an urgent CS is among the most dramatic anaesthetic events, requiring effective and vigilant services. It has been suggested that neonates should be delivered within 20 to 30 minutes after the decision of an urgent CS has been made ^3^. The time interval is, however, an extrapolation around time for the development of serious, life threatening acid base compromise ^4^. Various logistical programs aiming at improving the service have shown that time between decision and delivery can be reduced ^5^. The Swedish Society for Anaesthesia & Intensive Care has set recommendations that anaesthetic services should include an anaesthesiologist available within 5 minutes from the decision of an obstetric emergency and that an emergency CS incision should be possible to start within 15 minutes from the decision ^ii^. The explicit evidence to support a clear medical benefit of the 30 minute decision to delivery interval (DDI) limit is sparse and this may be more of a tool to use for auditing of anaesthesia services ^6^. The NICE guidelines extend category 2: Perform category 2 CS ^[2]^ in most situations within 75 minutes of making the decision ^iii^. It should also be acknowledged that the DDI is a composite end-point, including delay between obstetric decision, press the alarm button, time until start of anaesthesia, anaesthesia ready for surgery, and surgery time.

The primary aim of the present study was to assess the impact of anaesthetic technique and work shift on the DDI in emergency CS, with a decided DDI <30 minutes at our hospital, Danderyds Hospital (category 1 and emergency category 2 CS).

## Methods

The study was reviewed and approved by the Stockholm Ethical Review Board (reference number: 2016/825-31).

This is a retrospective chart review study using a proforma protocol; alarm logs and patient records for ECS at Danderyds Hospital from 1 ^st^ of January to October 31 ^st^ 2016 were collected and analysed. From the alarm logs, we assessed the time of the event and we then matched this information with the performed CS in our electronic surgical registration system (Orbit 5.7), from where we retrieved start time of anaesthesia, start and end of operation and type of anaesthesia performed. The follow up regarding foetal status and need of treatment was collected from the patient journal.

### Routines at the department

Danderyds Hospital is an emergency hospital with about 530 beds for general and gynaecological surgery, medicine and cardiac clinic, and includes two delivery departments with a total of 10800 deliveries/year. Two anaesthesia specialists and one anaesthesia registrar are in house on call for all anaesthesia services, including intensive care. There are at minimum three surgical teams, each including one anaesthesia nurse, one surgical nurse and one or two nursing assistants. One surgical team is located in and reserved for the women’s surgical department. In case of obstetric emergency collisions, a team from the general surgical department (located in the same building) reach the women’s department in 1–2 minutes to assist.

When the attending obstetrician decides on ECS in the most severe cases, needing immediate delivery, the alarm is pressed gathering the surgical team together with anaesthesia specialist, anaesthesia registrar and neonatologist. When the obstetrician estimates the ECS need a DDI within 30 minutes, the obstetrician first calls the anaesthesia specialist by phone to give a short report, including whether there is a well working epidural to top-up and then presses the alarm to gather the team. The obstetrician follows the patient to facilitate the process.

The OR is located central of the largest delivery department on the same floor and close to the women’s surgical department – within reach in 30–60 seconds. When occupied, an OR in the women’s surgical department is used.

When an ECS with an estimated DDI time >30 minutes is decided, the surgical team is gathered by phone and no alarm is used.

The anaesthesiologist specialist on call, responsible for the obstetric anaesthesia services decides on anaesthetic technique per set routines at the department. When the obstetrician urges for an immediate delivery general anaesthesia (GA) is recommended in most cases by our routines and with an explicit need for delivery within 30 minutes’ regional anaesthesia (RA) is recommended.

In the present study, only ECS needing the alarm, delivery immediately and up to 30 minutes, were studied, with the most common cause being sign of foetal distress.

No intervention or change of routine was initiated by or associated with the present study. GA was based on a rapid sequence induction with propofol and suxamethonium following pre-oxygenation and sevoflurane until umbilical cord is divided. Neuroaxial anaesthesia, spinal and top-up epidural followed standard routines. Spinal anaesthesia (SPA) with hyperbaric bupivacaine (approximately 2.4 ml 5 mg/ml), morphine (100 µg) and fentanyl (10 µg); top-up epidural (tEDA) with ropivacaine (7.5 mg/ml) and fentanyl (100 µg/20 ml ropivacaine) 15 – 20 ml.

### Statistical analysis

Descriptive statistics regarding the ECS and the variables in this study was made using mean, standard deviation (SD) and range, as well as median and interquartile range (IQR), as appropriate. Mann-Whitney U test was used for comparing means between two variables and Kruskal-Wallis H test was used when comparing two or more groups. Chi-square-test was used for test of differences between category data. A p-value <0.05 was considered significant and all data were analysed in IBM SPSS Statistics 23.

## Results

**Figure 1.**
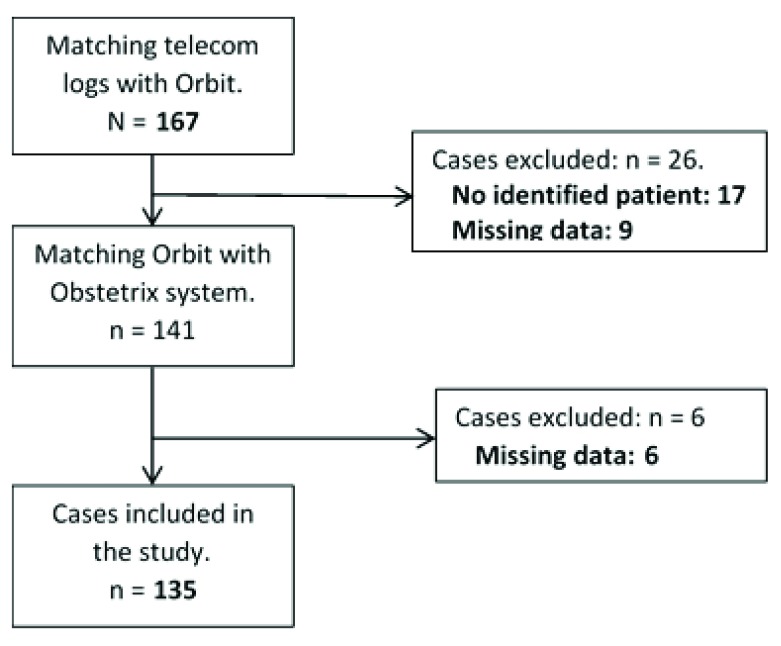
Patient inclusion.

During the study period, 150 ECS were identified from the record systems. Data for analysis was not retrieved in 15 cases, thus 135 ECS were included in the present analysis ( Figure 1).

The median DDI for the 135 ECS studied was 17 minutes (range, 5–41). The median DDI was significantly shorter for GA (10 minutes) compared to RA (SPA 20 minutes, tEDA 23 minutes) (p<0.0005; Table 1). The major time difference was in the time to establish adequate anaesthesia; GA shortened anaesthesia start to ready for surgery by 7 and 8 minutes as compared to SPA and tEDA, respectively. The time difference between SPA and tEDA was 1 minute: total 9 minutes (range, 1–16) and 10 minutes (range, 1–23), respectively ( Table 1).

**Table 1.** Time events for different anaesthetic techniques. Data are presented in minutes as median (range).

	Call to start anaesthesia	Start anaesthesia to ready for surgery	Surgery to delivery	DDI
GA (n = 64)	6 (1–17)	2 (1–8)	2 (1–4)	10 (5–21) ***
SPA (n = 30)	8 (1–23)	9 (1–16)	3 (1–7)	20 (13–33) ns.
tEDA (n = 41)	8 (1–25)	10 (1–23)	3 (1–8)	23 (12–41) ns.
*ALL (n =135)*	6 (1–25)	5 (1–23)	2 (1–8)	17 (5–41)

*** P < 0.0005 compared to reginal anaesthesia, ns. No significant difference between SPA and tEDA. GA, general anaesthesia; SPA, spinal anaesthesia; tEDA, top-up epidural anaesthesia; DDI, decision to delivery interval.

**Figure 2.**
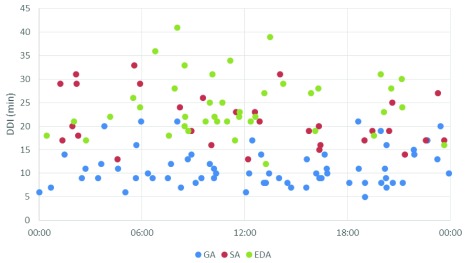
Decision to delivery interval for emergency caesarean sections studied (n=135) in relation to time of day. GA, general anaesthesia; SPA, spinal anaesthesia; tEDA, top-up epidural anaesthesia; DDI, decision to delivery interval.

There was no significant difference in DDI between different working shifts: daytime, on call during the week and weekends ( Table 2, Figure 2). Also, when divided into different time events, call to start of anaesthesia, start of anaesthesia to surgery, and surgery to delivery, no statistical difference between the different working shifts was found.

**Table 2.** Time events for the different work shifts: daytime, on-call during the week and at weekends. Data are presented in minutes as median (range).

	Call to start Anaesthesia	Start anaesthesia to ready for surgery	Surgery to delivery	DDI
Daytime (n = 37)	6 (1–25)	7 (1–23)	3 (1–6)	21 (6–41)
On-call (n = 60)	6 (1–23)	4 (1–19)	2 (1–7)	14.5 (6–36)
Weekend (n = 38)	9 (1–17)	6 (1–17)	2 (1–8)	18 (5–39)
*All (n = 135)*	6 (1–25)	5 (1–23)	2 (1–8)	17 (5–41)

DDI, decision to delivery interval.

Fourteen neonates had an Apgar score of <7 at 5 minutes: 11 out of the 64 mothers that received GA, 2 out of the 30 that received SA and 1 out of the 42 that received tEDA (p < 0.05). Seventeen neonates had a Ph < 7.1; *14 out of the 64 neonates with mothers having GA mothers, 2 neonates out of 30 mothers having spinal (DDI 15 and 17 minutes), and 1 out of the 41 mothers having t EDA (DDI 24 minutes).* In all, 39 neonates were transferred to the neonatal intensive care for further observation and treatment: 22, 10 and 7 had GA, SPA and tEDA, respectively (ns; Table 3).

**Table 3.** Comparison of anaesthetic technique and neonatal outcome.

Type of anaesthesia	GA (n = 64)	SPA (n = 30)	tEDA (n = 41)
**Apgar 5’,** median (IQR)	9 (3)	10 (2)	10 (1)
**Apgar 5’ <7,** n (%)	11 (17) *	2 (7)	1 (2)
**Umbilical cord arterial, mean**			
*pH*	*7.13*	*7.21*	*7.23*
*pCO _2_*	*9.07*	*7.40*	*7.28*
*Base excess*	*-9.53*	*-6.56*	*-6.55*
**CPAP,** n (%)	31 (48)	12 (40)	13 (31)
**Ventilation,** n (%)	26 (40)	13 (43)	6 (14)
**Intubation,** n (%)	5 (8)	1 (3)	1 (2)
**Neonatal unit,** n (%)	22 (34)	10 (33)	7 (17)

* p<0.05 Chi-square test, ns none significant between anaesthetic techniques GA, general anaesthesia; SPA, spinal anaesthesia; tEDA, top-up epidural anaesthesia; Apgar 5’, Apgar score at five minutes; CPAP, continuous positive airway pressure

Raw data for the present study Click here for additional data file. Copyright: © 2017 Hein A et al. 2017 Data associated with the article are available under the terms of the Creative Commons Zero "No rights reserved" data waiver (CC0 1.0 Public domain dedication).

## Discussion

Our study was designed as a quality audit of an important part of our anaesthesia service, providing effective anaesthesia for ECS. Our anaesthesia service was seemingly effective: work shift and day of the week did not impact the DDI. General anaesthesia was expectedly associated with the shortest time for anaesthesia, as well as the lowest DDI; however the DDI was kept within 30 minutes in a clear majority of cases also when spinal anaesthesia and top-up epidural anaesthesia were chosen. The conversion of an established labour epidural, increased time for anaesthesia and DDI, but only marginally. We did not find that the use of spinal anaesthesia or top-up epidural worsened neonate outcome. Thus, we do consider that our anaesthetic service is in line with national and local guidelines, since time to establish surgical anaesthesia was achieved in a timely fashion 24/7.

Time recommendations, such as a 30 minute DDI for ECS is more of a general recommendation than based on firm evidence. Anaesthesia for CS should always be managed on a benefit vs. risk basis. The degree of foetal and or maternal distress should form the basis for management and haste of delivery. One of our primary aims was to assess how our emergency obstetric anaesthesia service performed and thus an analysis of time lines was found to be a reasonable indicator. In 2006, Blom *et al.* published the results from a study assessing DDI in the US. Of the included 11,481 CS, 2,808 were performed for an emergency indication ^7^. Of these, 1,814 (65%) began within 30 minutes of the decision to operate, thus a lower figure than ours. Likewise, in a more recent meta-analyses, Tolcher *et al.* found that 79% of category 1 deliveries and 36% of category 2 deliveries were achieved within 30 minutes, with significantly shorter time in category 1 compared to category 2 deliveries ^8^. Thus, our service was found effective and “superior” to the results found in that study.

Time is of course not the key important variable; neonatal and parental outcome is without doubt the most important outcome. However, the aim of the present study was to assess logistics and the quality of the anaesthesia services. The proportion of neonates with a low 5 minute Apgar score was higher among the mothers that received GA. We interpret this finding as the result of the intra-uterine distress and not to the anaesthetic choice *per se*. We cannot, unfortunately, explicitly describe the degree of foetal distress, nor any further information around obstetric factors, placental ablatio, vaginal bleed, foetal Ph, etc. Blom *et al.* found that new-borns showing compromise, such as umbilical artery pH less than 7 and intubation in the delivery room, were significantly greater when the CS was commenced within 30 minutes, likely attesting to the need for emergent delivery. This is in line with our observations. Studies investigating neonatal outcomes correlated to DDI showed that there was a higher risk of overall 5-minute Apgar score < 7 and umbilical artery pH level <7.10 in cases involving shorter DDI. A study from Singapore published in 2016 showed results similar to ours; general anaesthesia was associated with a shorter DDI, but worse perinatal outcomes than regional anaesthesia ^9^. It must be acknowledged that this is a retrospective observational study. We did by no means intervene with what technique should be used. Anaesthesia was solely chosen by the anaesthetist on basis on the urgency for delivery.

We did not find any major difference in time delay between spinal anaesthesia, combining bupivacaine, fentanyl and morphine, and top-up epidural combining ropivacaine and fentanyl regarding time or neonatal outcome. Strouch *et al.* studied neonatal acid-base status and did not find any further acidosis associated to conversion epidural compared to spinal anaesthesia ^10^. We used a bupivacaine, morphine and fentanyl combination for the spinal anaesthesia, and ropivacaine and fentanyl for the epidural top-up. We used the 100-µg intrathecal dose morphine since it has been suggested to be an adequate balance between its benefits and side effects, pruritus and nausea/vomiting ^11^. Fentanyl facilitates onset ^12– 14^ and improves intraoperative analgesia. The addition of fentanyl for epidural anaesthesia has also been shown to improve quality of anaesthesia ^15, 16^. However, the intraoperative effect has been discussed for elective CS ^17^.

The conversion of a labour epidural to regional anaesthesia suitable for CS has been debated, but is today seemingly well-accepted practice ^18, 19^. The success rate for conversion is high; however prolonged duration of labour analgesia, repeated need of clinician administered bolus doses and obesity are factors suggested to increase the risk of failure ^20, 21^. Lidocaine with adrenalin with fentanyl supplementation is commonly used for conversion ^18^. Allam *et al.* showed carbonated lidocaine with adrenaline to be twice as fast as sole levo-bupivacaine to achieve a T5 touch/T4 cold block, when used for conversion ^22^. Carbonated local anaesthetics are not available in Sweden. A previous study comparing lidocaine/adrenaline/fentanyl to plain bupivacaine did not show significant difference in time to be ready for surgery ^23^. Sng *et al.* compared 2% lignocaine with adrenaline and fentanyl, 0.75% ropivacaine and 0.5% levo-bupivacaine for extension of low dose epidural analgesia for urgent CS and did not find any significant difference in time to reach surgical anaesthesia ^24^. We used ropivacaine and fentanyl mixture and achieved a rapid conversion.

The mother is exposed to increased anaesthetic risk when ECS is performed under general anaesthesia ^25^. Endler *et al.* suggested, following their review of maternal mortality in 1988, regional anaesthesia to be used when possible, avoiding the risk for serious airway complications ^26^. However, regional anaesthesia is not without risk ^27, 28^ and the Cochrane meta-analysis published in 2012 could not show any significant difference between general and regional anaesthesia in terms of risks ^29^.

The present results must be put into the perspective of the routine at our institution. When an epidural is in place during labour it is maintained with mid-wife administered bouluses (bupivacaine 1 mg/ml +sufentanil 0,5 µg/ml; 10 ml) on request of the mother, no background infusion is used. Midwifes are instructed to contact the anaesthesiologist without delay, if any decreased effect. When a push-button call is made by the obstetrician for a category 1 CS, we have always at least one experienced anaesthesiologist who is called together with anaesthesia and scrub nurses and a neonatologist. We have been working with the communication and process to facilitate regional anaesthesia when that is an option. The obstetrician informs the anaesthesiologist immediately after the decision if there is a labour epidural to top-up. The routine is to start the surgery activating dose already outside, but close to, the operation theatre when it seems appropriate, with continued supervision by the anaesthesiologist, when a well working labour epidural is in place and mother and child’s status allow. The theatre is in close proximity and can be reached within minutes. If a well working labour epidural is not in place a rapid spinal is chosen, if there is time. Warm Ringers lactate (1000 ml) is used as co-load and phenylephrine as first line to stabilize blood pressure to be combined with ephedrine where pulse is < 75 min ^-1^. During day time when the regular operation program is on-going there is always one room spared for emergent CS.

There are several limitations with our study. We have unfortunately not been able to discriminate absolute grade of emergency apart from the attending obstetricians’ decision of a DDI less than 30 minutes in the performed CS, due to our record keeping. The decision for anaesthetic technique may of course have been influenced by the degree of foetal and/or mother compromise. It is common practice to choose GA for the most urgent category 1 ECS, and to reserve spinal and epidural top-up for cases with less maternal or foetal compromise. We found a tendency of less pCO _2_, lower need of CPAP, ventilation and admission to neonatal unit in favour for EDA vs. GA and even vs. SA. Indeed, epidural top-up might have been chosen for the healthiest foetus. We did furthermore not explicitly study maternal effects, e.g. need for supplementation analgesia during regional anaesthesia. Consequently, further studies are warranted.

Terbutaline administration i.v. is common practice in our intrapartum intra-uterine resuscitation routine in case of asphyxia ECS, but we did not analyse the number of patients receiving tocolytics. Other possible factors are the occurrence of maternal hypotension events and amount consumed vasopressor, ephedrine and phenylephrine, which was not analysed in our study. Foetal heart rate monitoring is continued during the top-up epidural procedure and as foetal heart rate pattern is improved the need of urgency decreases and this might influence the DDI time.

In conclusion, we found our emergency obstetric anaesthesia service effective and adherent to guidelines for DDI. Anaesthesia for ECS must, however, always be based on an individual assessment, benefit vs. risk for mother and child. General anaesthesia was as expected associated with a more rapid DDI, but spinal anaesthesia with bupivacaine, morphine and fentanyl mixture, as well as top-up labour epidural provided similar rapid time to delivery and seems a reasonable benefit vs. risk option for category 1 ECS with acceptable DDI within 20–30 minutes. Further studies assessing effect on neonatal outcome associated with the choice of anaesthetic technique are warranted.

## Data availability

The data referenced by this article are under copyright with the following copyright statement: Copyright: © 2017 Hein A et al.

Data associated with the article are available under the terms of the Creative Commons Zero "No rights reserved" data waiver (CC0 1.0 Public domain dedication). 

Dataset 1: Raw data for the present study. doi, 10.5256/f1000research.13058.d183533 ^30^

## Foot notes

^i^ http://www.socialstyrelsen.se/nyheter/2014december/andelenkejsarsnittvarierarkraftigtilandet

^ii^ https://sfai.se/wp-content/uploads/files/11-4%20Obstetrisk_anestesi-och%20intensivv%C3%A5rd_organsation%20.pdf

^iii^ https://www.nice.org.uk/guidance/cg132/chapter/1-Guidance
