# Supplementary material for: The decision to delivery interval in emergency caesarean sections: Impact of anaesthetic technique and work shift
Source: F1000Res. 2017 Dec 11;6:1977. Originally published 2017 Nov 8. [Version 2] doi: 10.12688/f1000research.13058.2 (PMC5710389; doi:10.12688/f1000research.13058.2)
Supplement: Peer review history

# Peer review: Referee response for version 1

**DOI:** 10.5256/f1000research.14157.r28440

**Published:** 2017-11-30

**Authors:** Tülay Özkan Seyhan

**Affiliations:** 1 Department of Anesthesiology, İstanbul Faculty of Medicine, Istanbul University, Istanbul, Turkey

The manuscript titled “The decision to delivery interval in emergency caesarean sections: Impact of anaesthetic technique and work shift” is a well written study with limitations due to the retrospective nature.

 I have three comments for possible revision:

1. Please specify the top-up timing: The most interesting result as the similar onset of epidural top-up to spinal anesthesia without any given information about the used local anesthetic volume. When were top-ups injected? (in the delivery unit just following c-section decision or in OR?)
2. Please specify the type of spinal bupivacaine (hyper-/ iso-baric?)
3. The main limitation is the lack of details about cesarean delivery indications. It is not surprising that general anesthesia allowed to quickest possible operation start. But particularly general anesthesia group includes possibly more emergent cases leading to lower Apgar scores. It may give more insight to the reader, if the authors can give data about the number of neonates with pH<7.1 at birth.

I have read this submission. I believe that I have an appropriate level of expertise to confirm that it is of an acceptable scientific standard.

# Peer review: Referee response for version 1

**DOI:** 10.5256/f1000research.14157.r27757

**Published:** 2017-11-16

**Authors:** Antti J. Vaananen

**Affiliations:** 1 Department of Anesthesiology and Intensive Care, Helsinki University Central Hospital, Helsinki, Finland

This is a sound piece of work. Some points to consider regarding the article:

 Methods:

 The detailed description of the institutional procedures is a merit to this paper as it facilitates comparison of the results to other institutions.

 Were the parturients allocated into the different anesthesia groups according to the intended anesthesia type or final anesthesia type (is failed epidural top-up resulting in GA analyzed as epidural top-up or GA)? Was the rate of regional anesthesia failures addressed?

 Regarding epidural top-ups:

 The "start anesthesia to ready for surgery" is surprisingly fast for the epidural group given that ropivacaine (7.5 mg/ml) is used as the epidural anesthetic. It is stated in the paper that the top-up was initiated at the labour ward before transfer to the operating room which can be considered safe even in the absence of haemodynamic monitoring during transfer IF: a) the attending anesthesiologist is following the parturient to the operating room in these cases and b) the distance (=transfer time) from the labour room to the operating room is not long. 

 It would be helpful for the reader to see a brief description about the underlying labour analgesia system used (continuous infusion vs boluses on demand with or without background infusion) for the parturients with epidural catheters as this may have a major effect on the onset time of epidural top-up. 

 General comments:

 As the authors note in the discussion, it is evident that GA is more likely to be chosen in the more urgent cases (evident also as a 2 minutes lower median "call to start of anesthesia -time" and worse infant outcome parameters). This underlying difference in the obstetric urgency affects direct comparison of the anesthesia modes on outcome DDI times and could warrant presentation of the data in Table 2 separately for the GA cases as well as for the regional cases (even separately for all three anesthesia subgroups). 

 Ultimately this study provides important aspects in relation to the very similar overall DDI time when either spinal anesthesia or epidural anesthesia is employed (Table 1 and Figure 2) while showing that GA results in superior outcome DDI times - at least when obstetric emergency requires.

I have read this submission. I believe that I have an appropriate level of expertise to confirm that it is of an acceptable scientific standard.

# Response

**Published:** 2017-12-06

**Authors:** Jan Jakobsson

**Affiliations:** Department of Physiology and Pharmacology, Karolinska Institutet, Sweden

Dear Referees. Thank you for effective read and constructive and important comments. We have tried to amend the paper accordingly below are explicit responses to your comments. *Responses in Italics*

Methods:

The detailed description of the institutional procedures is a merit to this paper as it facilitates comparison of the results to other institutions.

Were the parturients allocated into the different anesthesia groups according to the intended anesthesia type or final anesthesia type (is failed epidural top-up resulting in GA analyzed as epidural top-up or GA)? Was the rate of regional anesthesia failures addressed?

*We did not have failure of blocks explicitly registered, thus we can’t give any exact data/details.*

Regarding epidural top-ups:

The "start anesthesia to ready for surgery" is surprisingly fast for the epidural group given that ropivacaine (7.5 mg/ml) is used as the epidural anesthetic. It is stated in the paper that the top-up was initiated at the labour ward before transfer to the operating room which can be considered safe even in the absence of haemodynamic monitoring during transfer IF: a) the attending anesthesiologist is following the parturient to the operating room in these cases

*Added to discussion,*

 and b) the distance (=transfer time) from the labour room to the operating room is not long. 

*Added in discussion,*

It would be helpful for the reader to see a brief description about the underlying labour analgesia system used

*Added in the discussion*

(continuous infusion vs boluses on demand with or without background infusion) for the parturients with epidural catheters as this may have a major effect on the onset time of epidural top-up. 

General comments:

As the authors note in the discussion, it is evident that GA is more likely to be chosen in the more urgent cases (evident also as a 2 minutes lower median "call to start of anesthesia -time" and worse infant outcome parameters). This underlying difference in the obstetric urgency affects direct comparison of the anesthesia modes on outcome DDI times and could warrant presentation of the data in Table 2 separately for the GA cases as well as for the regional cases (even separately for all three anesthesia subgroups). 

Ultimately this study provides important aspects in relation to the very similar overall DDI time when either spinal anesthesia or epidural anesthesia is employed (Table 1 and Figure 2) while showing that GA results in superior outcome DDI times - at least when obstetric emergency requires.

*Thank you for the adequate comment, the outcome is in our eyes overall positive and we the importance of no difference between spinal and top-up EDA*

The manuscript titled “The decision to delivery interval in emergency caesarean sections: Impact of anaesthetic technique and work shift” is a well written study with limitations due to the retrospective nature.

I have three comments for possible revision:

- Please specify the top-up timing: The most interesting result as the similar onset of epidural top-up to spinal anesthesia without any given information about the used local anesthetic volume.

*The mixture and volumes used are addressed in the method section, we can’t provide single patient data about volume/time.*

When were top-ups injected? (in the delivery unit just following c-section decision or in OR?)

*This is explicitly addressed and added to discussion *

- Please specify the type of spinal bupivacaine (hyper-/ iso-baric?)

*Hypebaric added* 

- The main limitation is the lack of details about cesarean delivery indications. It is not surprising that general anesthesia allowed to quickest possible operation start. But particularly general anesthesia group includes possibly more emergent cases leading to lower Apgar scores. It may give more insight to the reader, if the authors can give data about the number of neonates with pH<7.1 at birth.

*The aim was not to explicitly address neonatal outcome. We found that 14 out of the 64 neonates GA mothers had a Ph < 7.1, there was 2 neonates in spinal group of mothers (DDI 15 and 17 minutes), and only 1 out of the 41 mothers having top-up EDA (DDI 24 minutes). Added to the results.*
